# Increased sensitivity for negative emotional images in individuals with problematic pornography use

**DOI:** 10.3389/fpsyg.2024.1287455

**Published:** 2024-03-28

**Authors:** Shuangyi Qu, Ruiyu Li, Jianfeng Wang

**Affiliations:** ^1^School of Psychology, Chengdu Medical College, Chengdu, Sichuan, China; ^2^Sichuan Applied Psychology Research Center, Chengdu Medical College, Chengdu, Sichuan, China

**Keywords:** problematic pornography use, emotional processing, event-related potentials, emotion regulation, compulsive sexual behavior

## Abstract

**Background:**

Despite the frequent comorbidity of affective and addictive disorders, the significance of affective dysregulation in problematic pornography use (PPU) is commonly disregarded. The objective of this study is to investigate whether individuals with PPU demonstrate increased sensitivity to negative emotional stimuli in comparison to healthy controls (HCs).

**Methods:**

Electrophysiological responses were captured via event-related potentials (ERPs) from 27 individuals with PPU and 29 HCs. They completed an oddball task involving the presentation of deviant stimuli in the form of highly negative (HN), moderately negative (MN), and neutral images, with a standard stimulus being a neutral kettle image. To evaluate participants' subjective feelings of valence and arousal, the Self-Assessment Manikin (SAM) was employed.

**Results:**

Regarding subjective evaluations, individuals with PPU indicated diminished valence ratings for HN images as opposed to HCs. Concerning electrophysiological assessments, those with PPU manifested elevated N2 amplitudes in response to both HN and MN images when contrasted against neutral images. Additionally, PPU participants displayed an intensified P3 response to HN images in contrast to MN images, a distinction not evident within the HCs.

**Discussion:**

These outcomes suggest that individuals with PPU exhibited heightened reactivity toward negative stimuli. This increased sensitivity to negative cues could potentially play a role in the propensity of PPU individuals to resort to pornography as a coping mechanism for managing stress regulation.

## Introduction

High-speed internet connections and the proliferation of smartphones have contributed to the ease of consuming pornographic content. In a recent investigation conducted by Ballester-Arnal et al. (2021), findings reveal a range of prevalence rates concerning the lifetime engagement with pornography. Within the male demographic, the rates vary from 92% to 98%, while within females, the range spans from 50% to 91%. Predominantly, individuals consume pornography for entertainment purposes, with minimal adverse effects. However, a minority subgroup displays signs of problematic pornography use (PPU). This phenomenon is typified by intense impulses or cravings toward pornography, difficulties in regulating consumption, and sustained involvement despite unfavorable consequences (Wéry and Billieux, 2017). The categorization of PPU remains a subject of ongoing debate (Gola and Potenza, 2018; Kraus and Sweeney, 2019; Gola et al., 2022). PPU is commonly regarded as the most prevalent manifestation of Compulsive Sexual Behavior Disorder (CSBD) and is classified as an Impulse Control Disorder in ICD-11 (World Health Organization, 2019). Nevertheless, some scholars propose that defining PPU as a behavioral addiction characterized by core addictive components might be more appropriate (Kowalewska et al., 2018; Brand et al., 2020). Regardless of its classification, PPU shares phenomenological and potential neurobiological mechanisms with substance use disorders (Stark et al., 2018).

Numerous psychological frameworks concerning addictive and risky behaviors postulate the presence of an appetitive facet (associated with impulsivity) and/or a mood regulation facet (associated with anxiety) (Verheul et al., 1999; Raymond et al., 2003). For example, a wealth of evidence indicates that individuals with PPU exhibit heightened cue reactivity and craving for pornographic stimuli (Voon et al., 2014; Brand et al., 2016; Gola et al., 2017; Golec et al., 2021; Wang et al., 2021). Furthermore, some studies have also found that impulsivity and impaired response inhibition play a significant role in PPU (Antons and Brand, 2018; Seok and Sohn, 2020; Wang and Dai, 2020). However, it is notable that the predominant focus of current research has been directed toward the appetitive component of PPU, primarily investigating the influence of aberrant regulation within the brain's reward circuitry, particularly the ventral striatum, in driving sustained engagement in pornography consumption (Klein et al., 2022). Scarce attention has been dedicated to the examination of anxiety-related emotional regulatory elements within the context of PPU.

The comorbidity of mood and anxiety disorders with addictive behaviors has been extensively documented in clinical literature. Research in the domain of substance use disorders has indicated that individuals with substance addiction may display an excessive response to negative stimuli. This heightened sensitivity to negative emotions often drives them to resort to substances for stress relief (Grillon and Baas, 2003; Aguilar de Arcos et al., 2008). In a similar vein, the genesis of PPU could potentially be influenced by negative emotional states. For example, individuals with greater severity of PPU tended to experience elevated stress levels in their lives and acute affective responses related to anxiety (Antons et al., 2023). Moreover, research has indicated a connection between PPU/CSBD and anomalies in emotional regulation (Cashwell et al., 2017; Hashemi et al., 2018; Pepping et al., 2018). A recent study indicated that PPU was primarily influenced by difficulties in emotion regulation, loneliness, and gender (Cardoso et al., 2022). Compared to healthy individuals, those with CSBD tend to employ maladaptive strategies for emotional regulation more frequently (Engel et al., 2019). Furthermore, Bancroft and Vukadinovic (2004) have delineated three potential routes linking maladaptive negative emotions to CSBD: (1) utilizing sexual activity to fulfill regulatory goals during the experience of negative emotions; (2) employing sexual activity to shift attention away from external negative stimuli; and (3) engaging in sexual arousal as a conditioned response to intense negative emotions.

The aforementioned evidence suggests that heightened sensitivity to negative stimuli may contribute to individuals' overuse of pornography due to its stress-relief effects. However, to the best of our knowledge, there are currently no studies that have investigated the emotional processing sensitivity to negative stimuli in individuals with PPU. Therefore, the objective of this study is to investigate whether individuals with PPU demonstrate increased sensitivity to negative emotional stimuli in comparison to healthy individuals. In everyday life, emotional stimuli commonly exhibit variations in intensity. For instance, in the case of anger, its intensity can range from mild irritation to intense rage. Emotions of different intensities also exert distinct influences on cognitive processes (Li et al., 2008; Dixon-Gordon et al., 2015). As a result, this study incorporates two distinct intensity levels for negative emotional stimuli: moderately negative (MN) and highly negative (HN). Additionally, emotional processing typically encompasses intricate conscious and unconscious components (Kunaharan et al., 2017; Walla et al., 2017). Consequently, we concurrently measured participants' subjective and electrophysiological (event-related potentials, ERPs) responses to negative emotional stimuli.

ERPs are frequently employed as physiological markers for emotional reactions. Prior ERPs studies have revealed associations between emotional processing and both early (P2 and N2) and late (P3) components (Carretié et al., 2004; Delplanque et al., 2004, 2005). The frontal P2 and N2 components are considered to reflect early perceptual and attentional processes (Carretié et al., 2004; Delplanque et al., 2004). In contrast, the parietal P3 component is believed to mirror the elaborated processing of stimulus significance during later controlled processing stages (Hajcak and Foti, 2020). Past research consistently found that in healthy individuals, negative stimuli elicit larger amplitudes in these components compared to neutral stimuli (Hajcak et al., 2012). Therefore, the potential disparity in negative emotional sensitivity between individuals with PPU and their healthy counterparts might be reflected in these components. We employed the oddball task as a means of infrequently introducing emotional stimuli within an emotion-irrelevant cognitive context, thus simulating the occurrence of emotional events in natural settings (Delplanque et al., 2005). Moreover, this task has been shown to effectively elicit the above ERPs components (Olofsson and Polich, 2007).

We hypothesized that individuals with PPU would exhibit heightened negative emotional sensitivity in comparison to healthy individuals. Specifically, we anticipate that compared to healthy individuals, in PPU individuals, the following observations will be made: (1) Lower valence ratings for negative emotional stimuli; (2) Larger amplitude of ERPs for negative emotional stimuli; and (3) Differential responses to moderate and high negative emotional stimuli.

## Methods

### Participants

Using G^*^Power 3.1.9 (Faul et al., 2009), based on a medium effect size (f = 0.25), a power of 0.9, and a significance level of 0.05, the estimated sample size is 54. Therefore, the current study comprised 60 participants, selected from a previously established sample pool of 662 male college students. Four participants were excluded from the analysis due to excessive artifacts in the ERP recordings. The final sample consisted of 56 participants, with 29 individuals classified as healthy controls (HCs) and 27 as PPU participants (see Table 1). The research aligned with the ethical principles stipulated in the Declaration of Helsinki. Written informed consent was obtained from all participants. The research protocol was approved by the local Ethical Review Board.

**Table 1:** Demographic information and essential group characteristics.

**Variables**	**PPU participants (*n =* 27)**	**HCs (*n =* 29)**	**t/χ^2^**	***P***	**Cohen's d**
Age, *M* (*SD*)	20.26 (1.26)	20.10 (1.50)	0.42	0.676	0.11
SDS, *M* (*SD*)	38.59 (6.94)	33.55 (5.99)	2.92	**0.005**	0.78
SAS, *M* (*SD*)	39.56 (8.75)	34.17 (5.07)	2.79	**0.008**	0.76
PPCS, *M* (*SD*)	89.00 (8.45)	18.24 (0.58)	43.40	**< 0.001**	12.03
IGDS, *M* (*SD*)	15.33 (6.36)	14.55 (5.46)	0.50	0.623	0.13
PGSI, *M* (*SD*)	0.30 (0.87)	0.21 (0.68)	0.43	0.668	0.12

HCs, Healthy Controls; IGDS, Internet Gaming Disorder Scale; PGSI, Problem Gambling Severity Index; PPU, Problematic Pornography Use; PPCS, Problematic Pornography Consumption Scale; SAS, Self-Rating Anxiety Scale; SDS, Self-Rating Depression Scale. Bold values indicates statistical significance at the *p* < 0.05 level.

PPU was identified using the Problematic Pornography Consumption Scale (PPCS; Bothe et al., 2018). The PPCS offers a cutoff score of 76 (out of 126) to distinguish between problematic and non-problematic pornography use. Consequently, individuals scoring below 76 were categorized as HCs, while those scoring above 76 were categorized as PPU participants. All participants were above the age of 18 and met the following criteria: heterosexual orientation, right-handedness, absence of self-reported illicit drug usage, no presence of Axis-I psychiatric disorders (comprising major depressive disorder, anxiety disorder, and obsessive-compulsive disorder) based on assessment through the Mini International Neuropsychiatric Inventory (Sheehan et al., 1998). Additionally, participants did not have any current other behavioral addictions, such as gaming disorder (scoring below 36 on the nine-item short-form Internet Gaming Disorder Scale; IGDS9-SF) and gambling disorder (scoring below 8 on the nine-item Problem Gambling Severity Index; PGSI) (Ferris and Wynne, 2001; Pontes and Griffiths, 2015).

### Measurement instruments

#### PPU assessment

The PPCS comprises 18 items, designed to evaluate the fundamental aspects of addiction, encompassing salience, mood modification, conflict, tolerance, relapse, and withdrawal. Each of these components is assessed using a set of three items. Responses were rated on a 7-point scale, ranging from 1 (never) to 7 (all the time). The scale has good reliability and validity among Chinese samples (Chen et al., 2021). The Cronbach's α for the total scale in this study was 0.99.

#### Emotional assessment

The assessment of participants' levels of depression and anxiety is conducted through the implementation of the Zung Self-Rating Depression Scale (SDS; Zung et al., 1965) and the Zung Self-Rating Anxiety Scale (SAS; Zung, 1971). These two scales are designed to assess participants' self-perceived levels of depression and anxiety. Higher scores indicate more severe symptoms. Each scale comprises 20 items and responses were rated on a 4-point scale (1 = “none or a little time,” 4 = “most or all the time”). In this study, the Cronbach's α for SDS and SAS are 0.79 and 0.82, respectively.

#### Other behavioral addiction assessment

Gaming and gambling disorders are assessed using IGDS9-SF and PGSI respectively. The IGDS9-SF is a brief psychometric instrument derived from the nine fundamental criteria delineating gaming disorder as per DSM-5 guidelines. Participants' responses were assessed employing a 5-point Likert scale (1 = “Never,” 5 = “Very Often”). A score of 36 or above (out of a total of 45) on the assessment is indicative of potential gaming disorder. The PGSI is a questionnaire consisting of nine items that assess problematic gambling behavior. Responses are provided on a 4-point scale, ranging from 0 (Never) to 3 (Almost always). The sum of these item scores categorizes individuals: a total score of 0 signifies non-gamblers, 1–2 indicates low-risk gamblers, 3–7 suggests moderate-risk gamblers, and 8 or more signifies those with gambling disorder. In this study, the Cronbach's α for IGDS9-SF and PGSI are 0.91 and 0.66, respectively.

### Procedure

We utilized the survey platform Wenjuanxing (www.sojump.com) to disseminate screening questionnaires. The target participants consisted of individuals who had engaged in pornography use at least once within the previous 6 months. They were invited to participate and access the questionnaire by scanning a QR code for login purposes. In adherence to the aforementioned PPU screening criteria, individuals eligible for potential inclusion in the PPU group, as well as HCs, underwent a structured psychiatric interview administered by a psychiatrist. This interview aimed to rule out Axis I psychiatric disorders. Moreover, participants with a history of substance abuse or dependence were not present in the cohort. Only individuals satisfying the screening criteria and devoid of the previously mentioned disorders were extended invitations to partake in the subsequent ERPs study. Upon completion of the ERPs task, participants employed the Self-Assessment Manikin (SAM; Bradley and Lang, 1994) to assess their subjective reactions to each image along the dimensions of valence and arousal.

### Materials and tasks

#### Oddball task

We employed a modified oddball paradigm for our investigation, comprising four blocks, each containing 100 trials. In these trials, a ratio of 70% standard pictures to 30% deviant pictures was presented. The standard image depicted a kettle and remained consistent throughout the experiment. The selection of sixty deviant pictures was drawn from the Chinese Affective Picture System (Bai et al., 2005). These pictures were categorized into three conditions (neutral, MN, and HN), established based on valence and arousal norms. The average valence and arousal ratings were 5.55 and 2.89 for neutral pictures, 3.51 and 5.77 for MN pictures, and 1.91 and 7.04 for HN pictures, respectively. Within each block, ten images for each condition were presented, with each image repeated twice.

**Figure 1:**
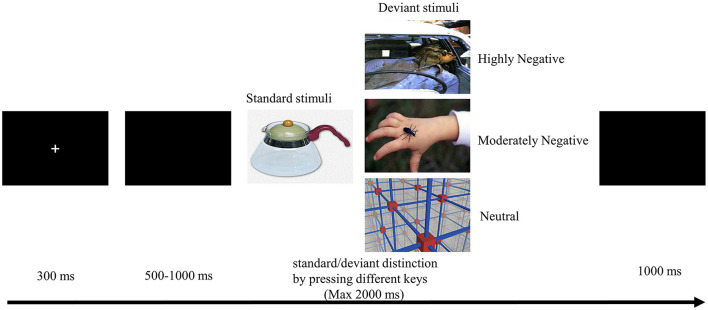
Experimental procedure and stimulus samples. Images reproduced with permission from the Chinese Affective Pictorial System (CAPS), Bai et al. (2005).

As depicted in Figure 1, each trial commenced with a 300 ms fixation cross, succeeded by a blank screen with variable duration (lasting between 500–1,000 ms). Subsequently, a 2,000 ms image was displayed, and participants were instructed to promptly and accurately respond by pressing the “F” key for standard stimuli or the “J” key for deviant stimuli. An intertrial interval of 1,000 ms was incorporated. The images were presented in a randomized sequence, and the assignment of response keys was counterbalanced across participants. Prior to the formal experiment, participants underwent 10 practice trials to acquaint themselves with the task.

#### SAM task

The SAM is a pictorial self-report scale commonly used to measure emotional responses and subjective feelings. The SAM consists of two pictorial scales: the valence scale and the arousal scale. Each scale employs a 9-point scale, where participants select a manikin that aligns with their emotional state. Valence ratings spanned from 1 (unpleasant) to 9 (pleasant), while arousal ratings ranged from 1 (relaxed) to 9 (aroused). The order of picture presentation was randomized across the emotion conditions.

### ERPs recording

Electroencephalographic (EEG) activity was captured through a 32-site elastic cap featuring active tin electrodes. The EEG signals were amplified using the BrainAmp amplifier (Brain Products GmbH, München, Germany). The reference electrodes were positioned on the left and right mastoids, and the ground electrode was situated on the medial frontal aspect. To monitor vertical electrooculograms (EOGs), the right eye was employed. Electrode impedance remained consistently below 5 kΩ. Subsequent offline data analysis was conducted utilizing Brain Vision Analyzer 2.0. Bandpass filtering was executed with parameters set at 0.01–30 Hz (24 dB/octave). Independent component analysis was undertaken to eliminate ocular artifacts.

To elicit ERPs, the EEG data corresponding to accurate responses within each condition were pooled and subjected to averaging. An epoch of 800 ms was employed, encompassing a 200 ms baseline interval preceding stimulus onset. Following a visual examination of the grand average ERP and drawing from prior research (e.g., Carretié et al., 2004; Delplanque et al., 2004; Prause et al., 2015; Wang et al., 2021), the P2 component was characterized by the mean amplitude within the 150–200 ms time window, the N2 component was defined by the mean amplitude within the 200–300 ms time span, and the P3 component was delineated by the mean amplitude within the 300–500 ms time range. Frontal P2 and N2 were evaluated at electrode sites F3, Fz, and F4, while parietal P3 was evaluated at electrode sites P3, Pz, and P4. This selection of electrodes aligns with prior investigations into the aforementioned components (e.g., Dai et al., 2011; Prause et al., 2015; Wang and Dai, 2020; Wang et al., 2021).

### Statistical analyses

The statistical analysis was executed through SPSS 22.0 software (SPSS, Chicago, USA). For questionnaire data, independent sample *t*-tests were utilized. Regarding behavioral measures (accuracy, reaction time (RT), and emotional ratings), a 2 × 3 repeated-measures analysis of variance (ANOVA) was employed.^1^ In this ANOVA, the factor Group (comprising PPU participants and HCs) was designated as a between-subjects factor, while Picture type (encompassing neutral, MN, and HN) was assigned as a within-subjects factor. For ERPs data (mean amplitudes of P2, N2, and P3), in addition to Group and Picture type, an additional within-subject factor Electrode was included. *Post hoc* analyses were conducted with Bonferroni pairwise comparisons. Greenhouse-Geisser correction was applied to account for degrees of freedom. Effect sizes were computed utilizing Cohen's d and partial eta squared ($\eta_{p}^{2}$).

## Results

### Questionnaire data

Table 1 provides descriptive statistics for the questionnaire data. As anticipated, individuals with PPU exhibited elevated scores on PPCS compared to HCs. Moreover, PPU participants demonstrated elevated levels of anxiety and depression relative to HCs. No significant differences were found between the two groups in terms of age, gaming symptoms, and gambling symptoms.

### Behavioral data: oddball task

Regarding RT, the analysis revealed a significant main effect of Picture type [*F*_(2, 108)_ = 4.70, *p* = 0.018, $\eta_{p}^{2}$ = 0.08]. Specifically, all participants exhibited notably quicker responses to HN pictures (585.24 ± 107.35 ms) compared to neutral pictures (609.03 ± 142.98 ms). However, there were no significant differences between MN (591.98 ± 115.03 ms) and neutral pictures, and between HN pictures as well. Furthermore, the main effect of Group did not reach significance [*F*_(1, 54)_ = 1.33, *p* = 0.254, $\eta_{p}^{2}$ = 0.02], and neither did the Group × Picture type interaction [*F*_(2, 108)_ = 0.02, *p* = 0.96]. No statistically significant variations emerged in accuracy (overall 96%).

### Behavioral data: SAM task

**Figure 2:**
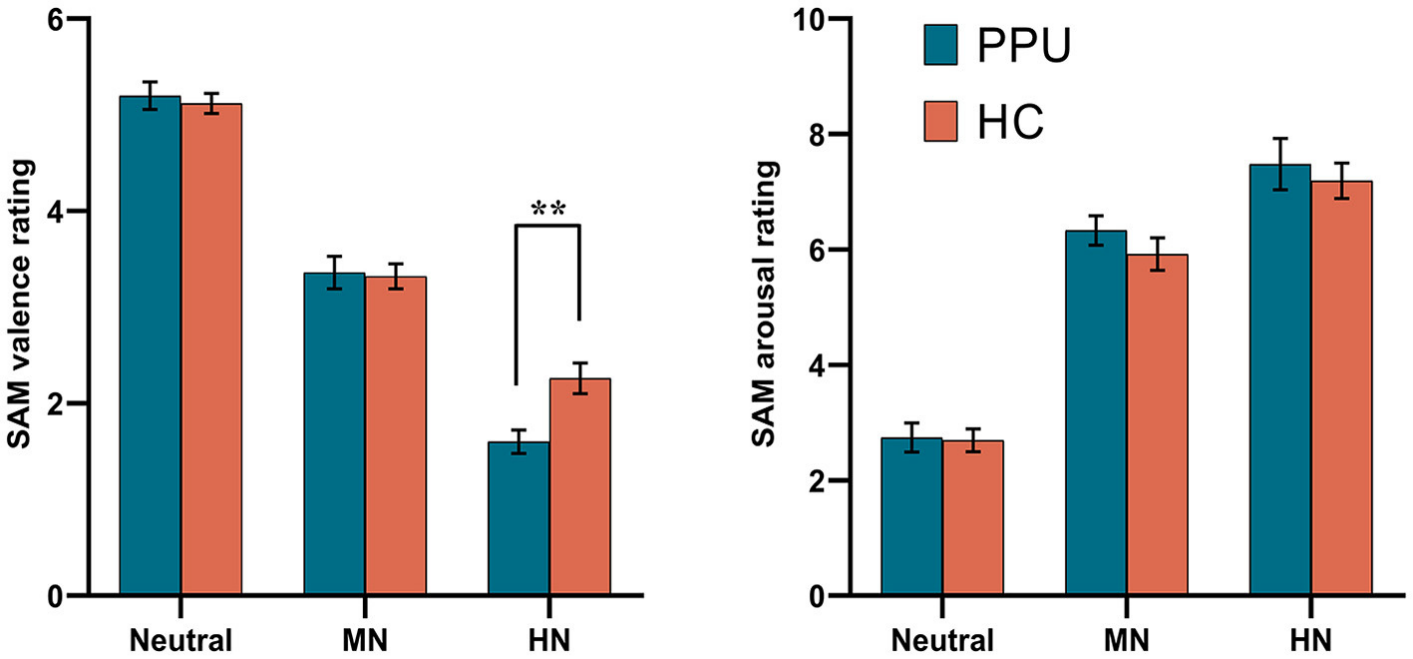
Self-Assessment Manikin (SAM) ratings by the three picture categories for PPU participants and HCs. Error bars represent one standard error. ^**^p < 0.01.

A significant main effect was observed for Picture type [*F*_(2, 108)_ = 299.68, *p* < 0.001, $\eta_{p}^{2}$ = 0.85] concerning valence ratings. HN pictures yielded notably lower pleasantness scores compared to MN pictures (*p* < 0.001), which, in turn, scored significantly lower than neutral pictures (*p* < 0.001). Additionally, a significant Group × Picture type interaction emerged, *F*_(2, 108)_ = 4.94, *p* = 0.011, $\eta_{p}^{2}$ = 0.08 (see Table 2 and Figure 2). Subsequent *post-hoc* analyses were conducted to explore potential intergroup variations within each emotional condition. Results indicated a significant group difference in the HN image condition, *t*_(54)_ = 3.23, *p* = 0.002, Cohen's d = 0.86, wherein PPU participants (1.60 ± 0.63) reported lower pleasantness scores than HCs (2.26 ± 0.86). However, group differences were not significant for MN images [*t*_(54)_ = −0.23, *p* > 0.82, Cohen's d = 0.06] and neutral images [*t*_(54)_ = −0.49, *p* > 0.62, Cohen's d = 0.13].

**Table 2:** Mean and standard deviations (in parentheses) of SAM ratings and ERPs amplitudes responses to the three picture categories for both PPU participants and HCs.

	**PPU participants (*****n*** **=** **27)**			**HCs (*****n*** **=** **29)**		
	**HN**	**MN**	**Neutral**	**HN**	**MN**	**Neutral**
Valence	1.60 (0.63)	3.37 (0.88)	5.20 (0.74)	2.26 (0.86)	3.32 (0.70)	5.12 (0.56)
Arousal	7.48 (2.31)	6.33 (1.33)	2.74 (1.30)	7.19 (1.65)	5.92 (1.52)	2.70 (1.07)
P2	−2.19 (4.48)	−3.18 (3.85)	−3.72 (3.46)	−3.33 (4.64)	−3.51 (4.07)	−3.42 (3.80)
N2	−10.55 (8.38)	−10.60 (9.36)	−7.89 (8.83)	−8.30 (5.80)	−8.12 (5.47)	−7.58 (6.45)
P3	6.55 (3.98)	4.58 (3.02)	3.05 (3.06)	4.71 (7.15)	4.99 (6.83)	2.12 (5.26)

ERPs, Event-related Potentials; HCs, Healthy Controls; HN, Highly Negative; MN, Moderately Negative; PPU, Problematic Pornography Use; SAM, Self-Assessment Manikin.

As for arousal ratings, only the main effect of Picture type reached significance, *F*_(2, 108)_ = 143.62, *p* < 0.001, $\eta_{p}^{2}$ = 0.73. HN pictures yielded notably higher arousal scores compared to MN pictures (*p* = 0.001), which, in turn, scored significantly higher than neutral pictures (*p* < 0.001).

### ERPs data

#### Frontal P2

Only the main effect of Electrode was significant [*F*_(2, 108)_ = 5.17, *p* = 0.013, $\eta_{p}^{2}$ = 0.09], with greater amplitude observed in the left hemisphere compared to the right hemisphere. Apart from this, neither the main effects of Group and Picture type nor the interaction between Group and Picture type were significant (*ps* > 0.08).

#### Frontal N2

**Figure 3:**
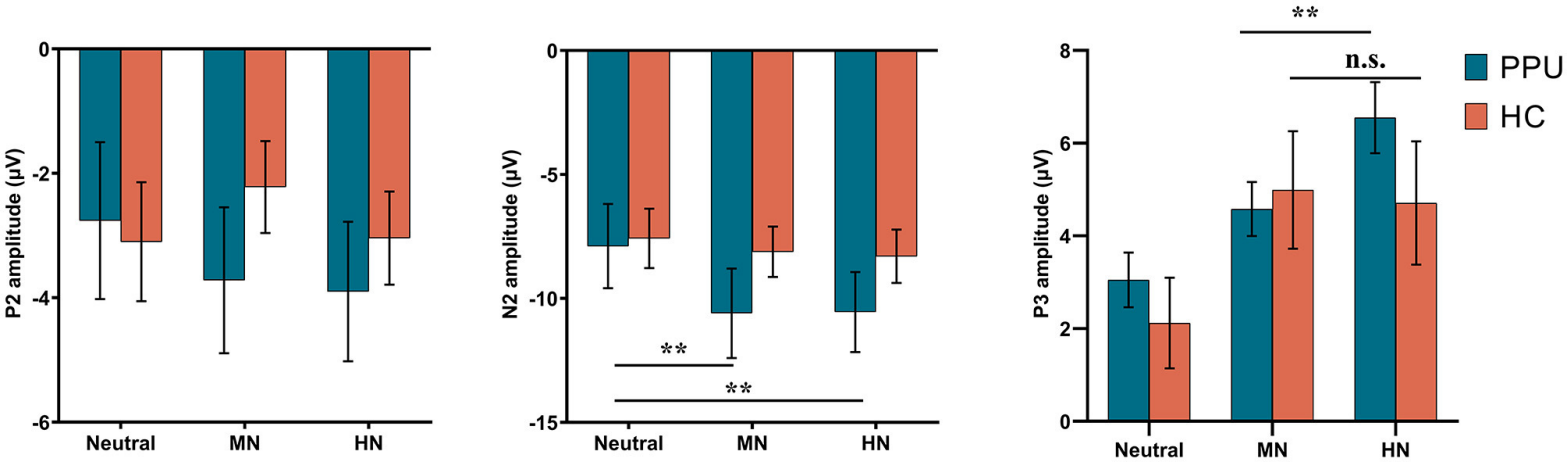
ERPs amplitudes by the three picture categories for PPU participants and HCs. Error bars represent one standard error. ^**^p < 0.01.

**Figure 4:**
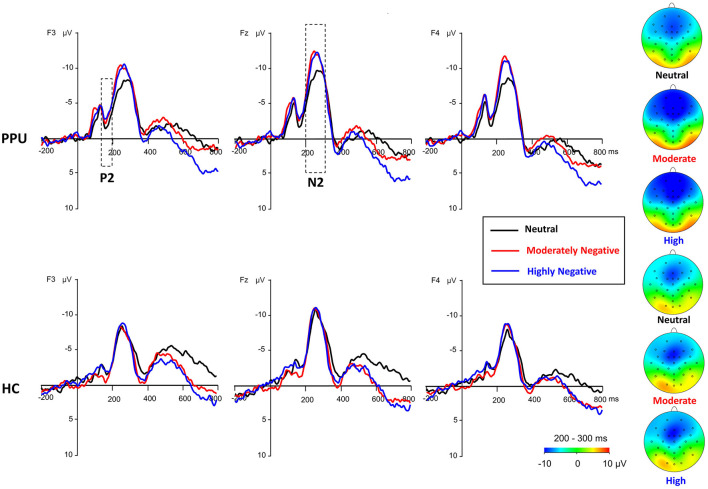
Waveforms and topography of the frontal P2 and N2 components for the PPU participants and HCs under the HN, MN, and neutral conditions for electrodes sites at F3, Fz, and F4. The N2 topography was calculated by averaging the data at 200–300 ms after picture onset.

We observed significant main effects of Picture type [*F*_(2, 108)_ = 10.32, *p* < 0.001, $\eta_{p}^{2}$ = 0.16] and Electrode [*F*_(2, 108)_ = 11.48, *p* < 0.001, $\eta_{p}^{2}$ = 0.18]. *Post hoc* analyses indicated that both HN (*p* = 0.002) and MN (*p* = 0.003) images elicited larger amplitude compared to neutral images, with no significant difference observed between the former two conditions (*p* = 1.00). Furthermore, electrode Fz elicited greater amplitude responses compared to F3 and F4, with no significant difference observed between the latter two. More importantly, a Group × Picture type interaction emerged, *F*_(2, 108)_ = 4.05, *p* = 0.027, $\eta_{p}^{2}$ = 0.07. A subsequent simple-effect analysis of Picture type revealed a significant main effect among PPU participants, *F*_(2, 52)_ = 12.13, *p* < 0.001, $\eta_{p}^{2}$ = 0.32, whereas such an effect was not observed among HCs, *F*_(2, 56)_ = 0.41, *p* > 0.40, $\eta_{p}^{2}$ = 0.03. *Post hoc* pairwise comparisons indicated that, for PPU participants, HN (*p* = 0.004) and MN (*p* = 0.001) images triggered greater N2 amplitudes compared to neutral images, although the former two did not exhibit a significant difference (*p* = 1.00) (see Figures 3, 4).

#### Parietal P3

**Figure 5:**
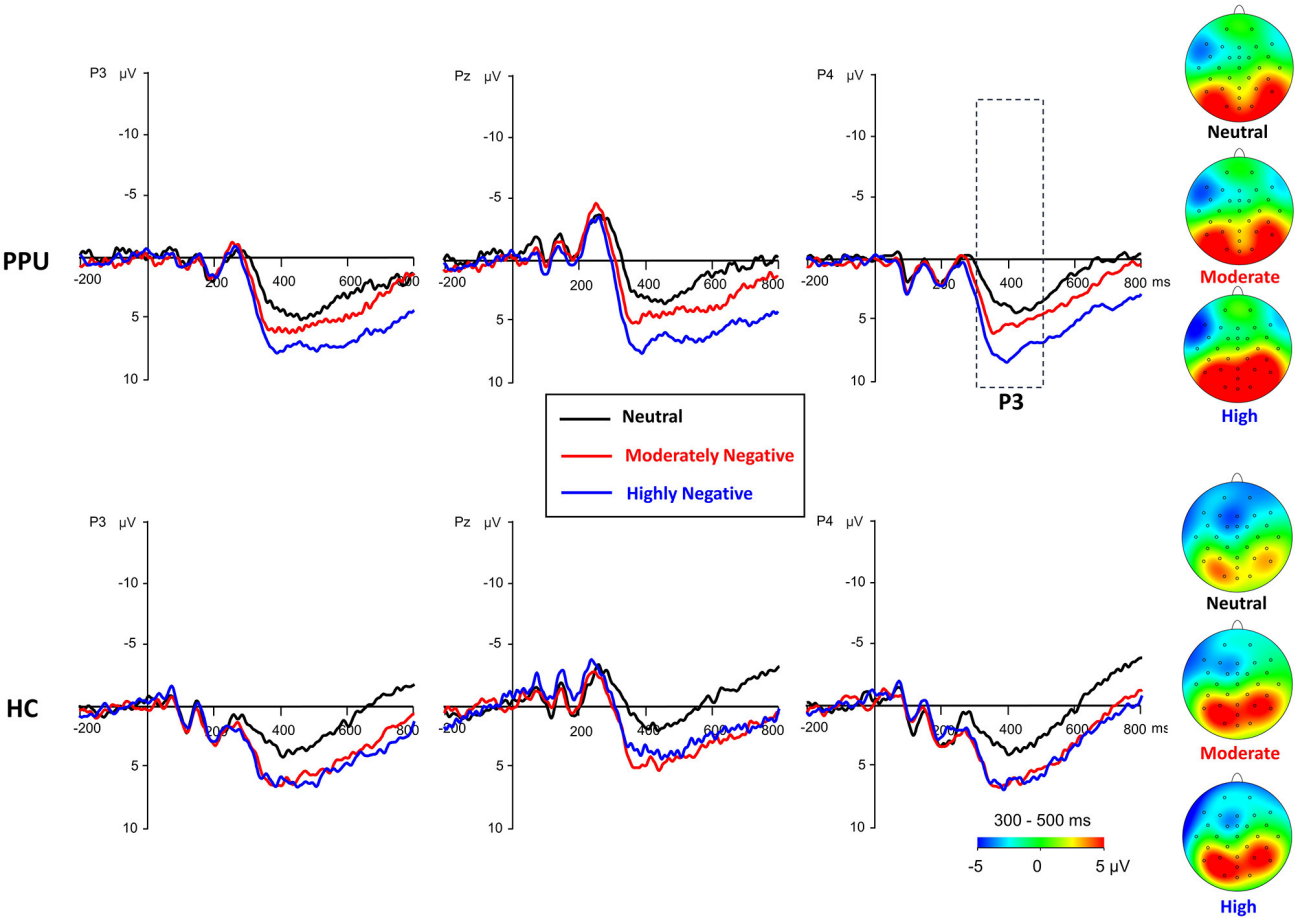
Waveforms and topography of the parietal P3 component for the PPU participants and HCs under the HN, MN, and neutral conditions for electrodes sites at P3, Pz, and P4. The P3 topography was calculated by averaging the data at 300–500 ms after picture onset.

The main effect of Picture type was significant, *F*_(2, 108)_ = 26.47, *p* < 0.001, $\eta_{p}^{2}$ = 0.33. HN (*p* < 0.001) and MN (*p* < 0.001) images evoked larger P3 amplitudes than neutral images, with no significant difference observed between the former two conditions (*p* = 0.064). The main effect of Electrode was significant, *F*_(2, 108)_ = 10.05, *p* < 0.001, $\eta_{p}^{2}$ = 0.16. Electrodes P3 and P4 elicited greater amplitude responses compared to Pz, with no significant difference observed between the former two. Importantly, the Group × Picture type interaction reached statistical significance, *F*_(2, 108)_ = 3.43, *p* = 0.036, $\eta_{p}^{2}$ = 0.06 (see Figures 3, 5). The subsequent simple-effect analysis of Picture type unveiled a significant main effect for both PPU participants [*F*_(2, 52)_ = 20.11, *p* < 0.001, $\eta_{p}^{2}$ = 0.44] and HCs [*F*_(2, 56)_ = 11.64, *p* < 0.001, $\eta_{p}^{2}$ = 0.29], with distinct patterns observed. Among PPU participants, HN images elicited larger P3 amplitudes than MN images (*p* = 0.005), which, in turn, evoked larger P3 amplitudes than neutral images (*p* = 0.019). In the case of HCs, both HN (*p* = 0.006) and MN (*p* = 0.001) images elicited larger P3 amplitudes than neutral images, and there was no statistically significant difference between the former two conditions (*p* = 1.00).

## Discussion

The primary aim of this study was to examine whether individuals with PPU exhibit distinct sensitivity to negative emotional stimuli compared to HCs. Taking into account both ERPs and subjective assessments, congruent results demonstrated heightened sensitivity to negative stimuli among PPU participants when compared to HCs. In the context of subjective assessments, PPU participants indicated reduced valence ratings for HN images in comparison to HCs. Turning to ERPs assessments, individuals with PPU exhibited increased N2 amplitudes for both HN and MN images when contrasted with neutral images. Moreover, while PPU participants demonstrated an amplified P3 response toward HN images when compared to MN images, this distinction was not observed within the HCs.

In this study, we discovered that during SAM assessments, participants with PPU assigned lower valence ratings to HN images compared to HCs. This subjective emotional assessment aligns with the electrophysiological findings. We observed that PPU individuals exhibited greater N2 amplitudes in response to HN and MN stimuli compared to neutral stimuli, in contrast to HCs. Frontal N2 activity observed during the oddball task is commonly associated with directing attention and maintaining vigilance toward unexpected salient and potentially harmful stimuli (Carretié et al., 2004; Clayson et al., 2012). These findings indicate that in the relatively early phases of emotional processing, participants with PPU devoted increased attentional resources to images displaying diverse levels of negative emotions. This phenomenon reflects their heightened responsiveness to negative stimuli. However, during the later stages of emotional processing, we observed that both PPU individuals and HCs exhibited larger P3 amplitudes in response to negative images (both HN and MN) compared to neutral images. The P3 component signifies cognitive assessment of stimulus significance, with larger amplitudes indicating heightened evaluation of stimulus meanings (Hajcak and Foti, 2020). As a result of the biological significance associated with negative stimuli, both groups demonstrated elevated responses to negative images during advanced cognitive stages. This response pattern could potentially mirror the human emotional tendency toward negative bias (Ito et al., 1998; Cacioppo and Gardner, 1999). However, it should be emphasized that this late-stage sensitivity to negative stimuli varies between PPU individuals and HCs. Specifically, PPU participants exhibit a more pronounced P3 amplitude response to HN compared to MN, whereas HCs show no difference in their response to negative stimuli of different intensities. This evidence, combined with the results of the N2 component, indicates that the heightened sensitivity to negative stimuli among PPU individuals is evident in both the early and late stages of emotional processing.

The current findings of heightened emotional reactivity toward unpleasant images are consistent with previous research in the domain of substance use disorders. Previous studies have established that individuals with substance use disorders tend to manifest an excessive response to negative stimuli (Grillon and Baas, 2003; Aguilar de Arcos et al., 2008). This increased sensitivity to negative stimuli often prompts individuals to resort to addictive substances as a means of alleviating adverse emotions (Cheetham et al., 2010). Models such as the tension reduction theory propose that potential addictive behaviors could regulate negative emotional states or mitigate stress through negative reinforcement mechanisms (Koob and Moal, 1997; Gola and Potenza, 2016). Similarly, the compensatory Internet use theory (Kardefelt-Winther, 2014) posits that certain individuals may turn to online environments as a coping strategy to alleviate negative emotions stemming from challenging life circumstances. This maladaptive coping mechanism might contribute to problematic usage or addiction. Consequently, individuals predisposed to heightened negative emotions face a higher susceptibility to addiction (Bonnaire and Baptista, 2019; Yuan et al., 2021). However, it should be noted that it is currently unclear whether the emotional regulation strategies utilized by individuals with PPU are intended to regulate negative emotions arising from the inability to access pornography or from other sources of stress. The negative reinforcement model suggests that the relationship between negative affect and addictive behavior may be bidirectional and cyclical (Kassel et al., 2007). Individuals experiencing higher levels of negative affect are at an elevated risk of using pornography as a coping mechanism (e.g., to enhance mood or divert attention from discomforting emotions). Subsequently, as dependence develops, individuals may persist in using pornography primarily to evade the negative emotional states linked to withdrawal.

Alternatively, the current findings could shed light on the personality traits of individuals with PPU or CSBD, characterized by mood and anxiety disorders (Lew-Starowicz et al., 2020). Previous studies have reported a high comorbidity rate between CSBD and mood or anxiety disorders (e.g., Raymond et al., 2003; Berberovic, 2013; Engel et al., 2019). A recent study further unveiled that men with CSBD exhibit maladaptive personality traits, including negative affect and detachment (Engel et al., 2023). In alignment with prior research, the present study disclosed that participants experiencing PPU demonstrated elevated levels of anxiety and depression when compared to HCs. Previous research has shown that individuals with anxiety or depression tend to display an augmented attentional bias toward negative stimuli, which significantly contributes to the maintenance and exacerbation of their anxiety or depressive states (Mogg and Bradley, 2005). Collectively, these findings suggest that heightened sensitivity to negative stimuli, coupled with resultant elevated anxiety or depression, may constitute foundational driving factors in the emergence of PPU symptoms.

This study holds significant theoretical and clinical importance. While the existing framework for understanding addictive behavior considers the presence of both appetitive and mood regulation components, prior research has primarily concentrated on the appetitive aspects associated with PPU. Even within the diagnostic criteria outlined in ICD-11, there is minimal emphasis on the influence of emotions. This study sheds light on the role of processing negative emotions in the context of PPU and contributes to a more comprehensive understanding of both PPU and CSBD. On the other hand, in clinical settings, individuals with PPU frequently grapple with an array of negative emotions, such as anxiety, depression, emptiness, and boredom. The findings of this study underscore the significance of mitigating their responsiveness to negative stimuli as a crucial facet of interventions and treatments targeting PPU.

This study is subject to several limitations. Firstly, the correlational nature of the research prevents the establishment of causal relationships. Therefore, it remains unclear whether sensitivity to negative stimuli precedes the existence of PPU or if PPU results in heightened sensitivity to negative stimuli. Nonetheless, a bidirectional causal relationship is plausible, wherein sensitivity to negative stimuli may serve as a precursor to PPU as a coping mechanism, while PPU, in turn, exacerbates maladaptive emotional processing. Secondly, the sample used in this study consisted of non-clinical undergraduate students. Future research should replicate these findings using clinical samples. Thirdly, given the higher prevalence of PPU among males (e.g., Grubbs et al., 2019), female participants were not included in this study. Thus, further investigation is necessary to determine the generalizability of the findings to the female population. Lastly, this study did not measure other personality traits (such as neuroticism, harm avoidance, or punishment sensitivity) that may be associated with cognitive processing bias. Future research should further control for these potential confounding variables.

## Data availability statement

The raw data supporting the conclusions of this article will be made available by the authors, without undue reservation.

## Ethics statement

The studies involving humans were approved by Chengdu Medical College Institutional Review Board. The studies were conducted in accordance with the local legislation and institutional requirements. The participants provided their written informed consent to participate in this study.

## Author contributions

SQ: Conceptualization, Data curation, Formal analysis, Investigation, Writing – original draft. RL: Conceptualization, Data curation, Formal analysis, Investigation, Writing – original draft. JW: Conceptualization, Supervision, Writing – review & editing.

## Footnotes

^1^Given notable disparities in anxiety and depression scores across groups, we performed an analysis of covariance (ANCOVA) on both behavioral and ERP data. Notably, the primary outcomes remained consistent despite the inclusion of these covariates. Please consult the Supplementary Material for a detailed presentation of the ANCOVA results.

## References

[B1] Aguilar de Arcos F. Verdejo-García A. Ceverino A. Montañez-Pareja M. López-Juárez E. Sánchez-Barrera M.. (2008). Dysregulation of emotional response in current and abstinent heroin users: negative heightening and positive blunting. Psychopharmacology 198, 159–166. 10.1007/s00213-008-1110-2 18330545

[B2] Antons S. Brand M. (2018). Trait and state impulsivity in males with tendency towards Internet-pornography-use disorder. Addict. Behav. 79, 171–177. 10.1016/j.addbeh.2017.12.029 29291508

[B3] Antons S. Büsche K. Mallon L. Wolf O. T. Diers M. Brand M. (2023). Stress susceptibility, affective responses toward acute stressors and emotion regulation strategies in the context of problematic pornography use. Sexual Health Compuls. 30, 231–251. 10.1080/26929953.2023.2220003

[B4] Bai L. Ma H. Huang Y. X. Luo Y. (2005). The development of native Chinese affective picture system-a pretest in 46 college students. Chinese Ment. Health J. 19, 719–722. 10.1016/j.molcatb.2005.02.001

[B5] Ballester-Arnal R. Castro-Calvo J. García-Barba M. Ruiz-Palomino E. Gil-Llario M. D. (2021). Problematic and non-problematic engagement in Online Sexual Activities across the lifespan. Comput. Hum. Behav. 120:106774. 10.1016/j.chb.2021.106774

[B6] Bancroft J. Vukadinovic Z. (2004). Sexual addiction, sexual compulsivity, sexual impulsivity, or what? Toward a theoretical model. J. Sex Res. 41, 225–234. 10.1080/00224490409552230 15497051

[B7] Berberovic D. (2013). Sexual compulsivity comorbidity with depression, anxiety, and substance use in students from Serbia and Bosnia and Herzegovina. Europe's J. Psychol. 9, 517–530. 10.5964/ejop.v9i3.595

[B8] Bonnaire C. Baptista D. (2019). Internet gaming disorder in male and female young adults: The role of alexithymia, depression, anxiety and gaming type. Psychiat. Res. 272, 521–530. 10.1016/j.psychres.2018.12.158 30616119

[B9] Bothe B. Tóth-Király I. Zsila Á. Griffiths M. D. Demetrovics Z. Orosz G. (2018). The development of the problematic pornography consumption scale (PPCS). J. Sex Res. 55, 395–406. 10.1080/00224499.2017.1291798 28276929

[B10] Bradley M. M. Lang P. J. (1994). Measuring emotion: the self-assessment manikin and the semantic differential. J. Behav. Ther. Exper. Psychiat. 25, 49–59. 10.1016/0005-7916(94)90063-9 7962581

[B11] Brand M. Rumpf H. J. Demetrovics Z. MÜller A. Stark R. King D. L.. (2020). Which conditions should be considered as disorders in the International Classification of Diseases (ICD-11) designation of “other specified disorders due to addictive behaviors”? J. Behav. Addict. 11, 150–159. 10.1556/2006.2020.00035 32634114 PMC9295220

[B12] Brand M. Snagowski J. Laier C. Maderwald S. (2016). Ventral striatum activity when watching preferred pornographic pictures is correlated with symptoms of Internet pornography addiction. NeuroImage 129, 224–232. 10.1016/j.neuroimage.2016.01.033 26803060

[B13] Cacioppo J. T. Gardner W. L. (1999). Emotion. Ann. Rev. Psychol. 50, 191–214. 10.1146/annurev.psych.50.1.191 10074678

[B14] Cardoso J. Ramos C. Brito J. Almeida T. C. (2022). Predictors of pornography use: difficulties in emotion regulation and loneliness. J. Sexual Med. 19, 620–628. 10.1016/j.jsxm.2022.01.005 35165051

[B15] Carretié L. Hinojosa J. A. Martín-Loeches M. Mercado F. Tapia M. (2004). Automatic attention to emotional stimuli: neural correlates. Hum. Brain Mapp. 22, 290–299. 10.1002/hbm.20037 15202107 PMC6871850

[B16] Cashwell C. S. Giordano A. L. King K. Lankford C. Henson R. K. (2017). Emotion regulation and sex addiction among college students. Int. J. Mental Health Addict. 15, 16–27. 10.1007/s11469-016-9646-6

[B17] Cheetham A. Allen N. B. Yücel M. Lubman D. I. (2010). The role of affective dysregulation in drug addiction. Clin. Psychol. Rev. 30, 621–634. 10.1016/j.cpr.2010.04.005 20546986

[B18] Chen L. Luo X. Bothe B. Jiang X. Demetrovics Z. Potenza M. N. (2021). Properties of the problematic pornography consumption scale (PPCS-18) in community and subclinical samples in China and Hungary. Addict. Behav. 112:106591. 10.1016/j.addbeh.2020.106591 32768797

[B19] Clayson P. E. Clawson A. Larson M. J. (2012). The effects of induced state negative affect on performance monitoring processes. Soc. Cogn. Affect. Neurosci. 7, 677–688. 10.1093/scan/nsr040 21685443 PMC3427863

[B20] Dai S. Y. Ma Q. G. Wang X. Y. (2011). Attentional bias to addiction-related stimuli in internet addiction patients: an ERP study. J. Psychol. Sci. 34, 1302–1307. 10.1007/s11769-011-0450-8

[B21] Delplanque S. Lavoie M. E. Hot P. Silvert L. Sequeira H. (2004). Modulation of cognitive processing by emotional valence studied through event-related potentials in humans. Neurosci. Lett. 356, 1–4. 10.1016/j.neulet.2003.10.014 14746887

[B22] Delplanque S. Silvert L. Hot P. Sequeira H. (2005). Event-related P3a and P3b in response to unpredictable emotional stimuli. Biol. Psychol. 68, 107–120. 10.1016/j.biopsycho.2004.04.006 15450691

[B23] Dixon-Gordon K. L. Aldao A. De Los Reyes A. (2015). Emotion regulation in context: Examining the spontaneous use of strategies across emotional intensity and type of emotion. Person. Indiv. Differ. 86, 271–276. 10.1016/j.paid.2015.06.011

[B24] Engel J. Carstensen M. Veit M. Sinke C. Kneer J. Hartmann U.. (2023). Personality dimensions of compulsive sexual behavior in the Sex@ Brain study. J. Behav. Addict. 12, 408–420. 10.1556/2006.2023.00029 37384566 PMC10316167

[B25] Engel J. Veit M. Sinke C. Heitland I. Kneer J. Hillemacher T.. (2019). Same same but different: a clinical characterization of men with hypersexual disorder in the sex@ brain study. J. Clin. Med. 8:157. 10.3390/jcm8020157 30704084 PMC6406591

[B26] Faul F. Erdfelder E. Buchner A. Lang A. G. (2009). Statistical power analyses using G^*^ Power 3.1: tests for correlation and regression analyses. Behav. Res. Methods 41, 1149–1160. 10.3758/BRM.41.4.1149 19897823

[B27] Ferris J. A. Wynne H. J. (2001). The Canadian Problem Gambling Index. Ottawa, ON: Canadian Centre on substance abuse, 1–59.

[B28] Gola M. Lewczuk K. Potenza M. N. Kingston D. A. Grubbs J. B. Stark R.. (2022). What should be included in the criteria for compulsive sexual behavior disorder? J. Behav. Addict. 11, 160–165. 10.1556/2006.2020.00090 34329192 PMC9295236

[B29] Gola M. Potenza M. N. (2016). Paroxetine treatment of problematic pornography use: a case series. J. Behav. Addict. 5, 529–532. 10.1556/2006.5.2016.046 27440474 PMC5264421

[B30] Gola M. Potenza M. N. (2018). Promoting educational, classification, treatment, and policy initiatives: Commentary on: Compulsive sexual behaviour disorder in the ICD-11 (Kraus et al., 2018). J. Behav. Addict. 7, 208–210. 10.1556/2006.7.2018.51 29895182 PMC6174588

[B31] Gola M. Wordecha M. Sescousse G. Lew-Starowicz M. Kossowski B. Wypych M.. (2017). Can pornography be addictive? An fMRI study of men seeking treatment for problematic pornography use. Neuropsychopharmacology 42, 2021–2031. 10.1038/npp.2017.78 28409565 PMC5561346

[B32] Golec K. Draps M. Stark R. Pluta A. Gola M. (2021). Aberrant orbitofrontal cortex reactivity to erotic cues in compulsive sexual behavior disorder. J. Behav. Addict. 10, 646–656. 10.1556/2006.2021.00051 34437297 PMC8997235

[B33] Grillon C. Baas J. (2003). A review of the modulation of the startle reflex by affective states and its application in psychiatry. Clin. Neurophysiol. 114, 1557–1579. 10.1016/S1388-2457(03)00202-5 12948786

[B34] Grubbs J. B. Kraus S. W. Perry S. L. (2019). Self-reported addiction to pornography in a nationally representative sample: the roles of use habits, religiousness, and moral incongruence. J. Behav. Addict. 8, 88–93. 10.1556/2006.7.2018.134 30632378 PMC7044607

[B35] Hajcak G. Foti D. (2020). Significance? Significance! Empirical, methodological, and theoretical connections between the late positive potential and P300 as neural responses to stimulus significance: an integrative review. Psychophysiology 57:e13570. 10.1111/psyp.13570 32243623

[B36] Hajcak G. Weinberg A. MacNamara A. Foti D. (2012). “ERPs and the study of emotion,” in The Oxford Handbook of Event-Related Potential Components, eds. S. J. Luck and E. S. Kappenman (Oxford: Oxford University Press), 441–472. 10.1093/oxfordhb/9780195374148.013.0222

[B37] Hashemi S. G. S. Shalchi B. Yaghoubi H. (2018). Difficulties in emotion regulation, psychological well-being, and hypersexuality in patients with substance use disorder in Iran. Iranian J. Psychiat. Behav. Sci. 12:e10449. 10.5812/ijpbs.10449

[B38] Ito T. A. Larsen J. T. Smith N. K. Cacioppo J. T. (1998). Negative information weighs more heavily on the brain: the negativity bias in evaluative categorizations. J. Person. Soc. Psychol. 75, 887–900. 10.1037/0022-3514.75.4.887 9825526

[B39] Kardefelt-Winther D. (2014). A conceptual and methodological critique of internet addiction research: towards a model of compensatory internet use. Comput. Hum. Behav. 31, 351–354. 10.1016/j.chb.2013.10.059

[B40] Kassel J. D. Veilleux J. C. Wardle M. C. Yates M. C. Greenstein J. E. Evatt D. P.. (2007). “Negative affect and addiction,” in Stress and addiction: Biological and Psychological Mechanisms, ed. M. al Absi (London: Academic Press). 10.1016/B978-012370632-4/50011-5

[B41] Klein S. Krikova K. Antons S. Brand M. Klucken T. Stark R. (2022). Reward responsiveness, learning, and valuation implicated in problematic pornography use—a research domain criteria perspective. Curr. Addict. Rep. 9, 114–125. 10.1007/s40429-022-00423-w

[B42] Koob G. F. Moal M. L. (1997). Drug abuse: hedonic homeostatic dysregulation. Science 278, 52–58. 10.1126/science.278.5335.52 9311926

[B43] Kowalewska E. Grubbs J. B. Potenza M. N. Gola M. Draps M. Kraus S. W. (2018). Neurocognitive mechanisms in compulsive sexual behavior disorder. Curr. Sexual Health Rep. 10, 255–264. 10.1007/s11930-018-0176-z

[B44] Kraus S. W. Sweeney P. J. (2019). Hitting the target: Considerations for differential diagnosis when treating individuals for problematic use of pornography. Arch. Sexual Behav. 48, 431–435. 10.1007/s10508-018-1301-9 30229519

[B45] Kunaharan S. Halpin S. Sitharthan T. Bosshard S. Walla P. (2017). Conscious and non-conscious measures of emotion: Do they vary with frequency of pornography use? Appl. Sci. 7:493. 10.3390/app7050493

[B46] Lew-Starowicz M. Lewczuk K. Nowakowska I. Kraus S. Gola M. (2020). Compulsive sexual behavior and dysregulation of emotion. Sexual Med. Rev. 8, 191–205. 10.1016/j.sxmr.2019.10.003 31813820

[B47] Li H. Yuan J. Lin C. (2008). The neural mechanism underlying the female advantage in identifying negative emotions: an event-related potential study. Neuroimage 40, 1921–1929. 10.1016/j.neuroimage.2008.01.033 18343686

[B48] Mogg K. Bradley B. P. (2005). Attentional bias in generalized anxiety disorder versus depressive disorder. Cogn. Ther. Res. 29, 29–45. 10.1007/s10608-005-1646-y

[B49] Olofsson J. K. Polich J. (2007). Affective visual event-related potentials: arousal, repetition, and time-on-task. Biol. Psychol. 75, 101–108. 10.1016/j.biopsycho.2006.12.006 17275979 PMC1885422

[B50] Pepping C. A. Cronin T. J. Lyons A. Caldwell J. G. (2018). The effects of mindfulness on sexual outcomes: the role of emotion regulation. Arch. Sexual Behav. 47, 1601–1612. 10.1007/s10508-017-1127-x 29453643

[B51] Pontes H. M. Griffiths M. D. (2015). Measuring DSM-5 internet gaming disorder: development and validation of a short psychometric scale. Comput. Hum. Behav. 45, 137–143. 10.1016/j.chb.2014.12.006

[B52] Prause N. Steele V. R. Staley C. Sabatinelli D. Hajcak G. (2015). Modulation of late positive potentials by sexual images in problem users and controls inconsistent with “porn addiction”. Biol. Psychol. 109, 192–199. 10.1016/j.biopsycho.2015.06.005 26095441

[B53] Raymond N. C. Coleman E. Miner M. H. (2003). Psychiatric comorbidity and compulsive/impulsive traits in compulsive sexual behavior. Compr. Psychiat. 44, 370–380. 10.1016/S0010-440X(03)00110-X 14505297

[B54] Seok J. W. Sohn J. H. (2020). Response inhibition during processing of sexual stimuli in males with problematic hypersexual behavior. J. Behav. Addict. 9, 71–82. 10.1556/2006.2020.00003 32359232 PMC8935199

[B55] Sheehan D. V. Lecrubier Y. Sheehan K. H. Amorim P. Janavs J. Weiller E.. (1998). The mini-international neuropsychiatric interview (MINI): the development and validation of a structured diagnostic psychiatric interview for DSM-IV and ICD-10. J. Clin. Psychiat. 59, 22–33. 10.1037/t18597-000 9881538

[B56] Stark R. Klucken T. Potenza M. N. Brand M. Strahler J. (2018). A current understanding of the behavioral neuroscience of compulsive sexual behavior disorder and problematic pornography use. Curr. Behav. Neurosci. Rep. 5, 218–231. 10.1007/s40473-018-0162-9

[B57] Verheul R. van den Brink W. Geerlings P. E. T. E. R. (1999). A three-pathway psychobiological model of craving for alcohol. Alcohol Alcohol. 34, 197–222. 10.1093/alcalc/34.2.197 10344781

[B58] Voon V. Mole T. B. Banca P. Porter L. Morris L. Mitchell S.. (2014). Neural correlates of sexual cue reactivity in individuals with and without compulsive sexual behaviours. PloS ONE 9:e102419. 10.1371/journal.pone.0102419 25013940 PMC4094516

[B59] Walla P. Koller M. Brenner G. Bosshard S. (2017). Evaluative conditioning of established brands: Implicit measures reveal other effects than explicit measures. J. Neurosci. Psychol. Econ. 10, 24–41. 10.1037/npe0000067

[B60] Wang J. Chen Y. Zhang H. (2021). Electrophysiological evidence of enhanced early attentional bias toward sexual images in individuals with tendencies toward cybersex addiction. J. Behav. Addict. 10, 1036–1047. 10.1556/2006.2021.00082 34817398 PMC8987427

[B61] Wang J. Dai B. (2020). Event-related potentials in a two-choice oddball task of impaired behavioral inhibitory control among males with tendencies towards cybersex addiction. J. Behav. Addict. 9, 785–796. 10.1556/2006.2020.00059 32903206 PMC8943673

[B62] Wéry A. Billieux J. (2017). Problematic cybersex: conceptualization, assessment, and treatment. Addict. Behav. 64, 238–246. 10.1016/j.addbeh.2015.11.007 26646983

[B63] World Health Organization (2019). International Classification of Diseases (ICD) 11th Revision. Available online at: https://icd.who.int/en (Retrieved March 22, 2024).

[B64] Yuan G. Elhai J. D. Hall B. J. (2021). The influence of depressive symptoms and fear of missing out on severity of problematic smartphone use and Internet gaming disorder among Chinese young adults: a three-wave mediation model. Addict. Behav. 112:106648. 10.1016/j.addbeh.2020.106648 32977268

[B65] Zung W. W. (1971). A rating instrument for anxiety disorders. Psychosomatics 12, 371–379. 10.1016/S0033-3182(71)71479-0 5172928

[B66] Zung W. W. Richards C. B. Short M. J. (1965). Self-rating depression scale in an outpatient clinic: further validation of the SDS. Arch. General Psychiat. 13, 508–515. 10.1001/archpsyc.1965.01730060026004 4378854

