# Supplementary material for: Increased sensitivity for negative emotional images in individuals with problematic pornography use
Source: Front Psychol. 2024 Mar 28;15:1287455. doi: 10.3389/fpsyg.2024.1287455 (PMC11007122; doi:10.3389/fpsyg.2024.1287455)
Supplement: Supplementary file 1 [file Table_1.DOCX]

**Supplementary Materials**

Table S1. Covariate analysis of covariance (ANCOVA) results for SAM ratings and ERPs components with anxiety and depression as covariates

| Dependent Variable | Group | Picture type | Group × Picture type |
| --- | --- | --- | --- |
| Valence | *F*(1, 52)=4.03, *P*=0.050, η=0.07 | *F*(2, 104)=10.17, *P<*0.001, η=0.16 | *F*(2, 104)=3.59, *P=*0.035, η=0.07 |
| Arousal | *F*(1, 52)=0.04, *P=*0.846, η=0.00 | *F*(2, 104)=8.91, *P=*0.002, η=0.15 | *F*(2, 104)=0.75, *P=*0.425, η=0.01 |
| P2 | *F*(1, 52)=0.28, *P=*0.597, η=0.01 | *F*(2, 104)=0.66, *P=*0.493, η=0.01 | *F*(2, 104)=3.04, *P=*0.062, η=0.05 |
| P3 | *F*(1, 52)=0.63, *P=*0.431, η=0.01 | *F*(2, 104)=0.20, *P=*0.795, η=0.00 | *F*(2, 104)=3.32, *P=*0.045, η=0.06 |
| N2 | *F*(2, 104)=0.46, *P=*0.502, η=0.01 | *F*(2, 104)=3.02, *P=*0.063, η=0.06 | *F*(2, 104)=5.28, *P=*0.010, η=0.09 |
| RT | *F*(1, 52)=0.06, *P=*0.80, η=0.001 | *F*(2, 104)=0.00, *P=*1.00, η=0.000 | *F*(2, 104)=0.00, *P=*0.99, η=0.000 |
